# Clinical efficacy of Tailin formulation combined with continuous low-dose antimicrobial therapy for recurrent urinary tract infection: study protocol for a multicenter, double-blind, randomized, controlled clinical trial

**DOI:** 10.1186/s13063-021-05830-4

**Published:** 2021-12-11

**Authors:** Tonglu Li, Yingru Xu, Xuezhong Gong

**Affiliations:** Department of Nephrology, Shanghai Municipal Hospital of Traditional Chinese Medicine, Shanghai University of Traditional Chinese Medicine, 274 Zhijiang Middle Road, Shanghai, 200071 China

**Keywords:** Tailin formulation, Recurrent urinary tract infection (rUTI), Continuous low-dose antibiotic therapy (CLAT), Traditional Chinese medicine (TCM), Randomized controlled trial (RCT)

## Abstract

**Background:**

Given the increasing rates of antimicrobial resistance (AMR), recurrent urinary tract infection (rUTI) is becoming refractory more and more. Antibiotic prophylaxis including continuous low-dose antibiotic therapy (CLAT), is the common treatment for rUTI of the world. However, the presumably adverse reactions caused by CLAT alone should be paid more attention. Studies indicated that Chinese herbal medicine (CHM) might be an available treatment method for rUTI. Tailin formulation (TLF) is a herbal prescription developed for the treatment of rUTI in the 2000s in Shanghai Municipal Hospital of Traditional Chinese Medicine. Our previous studies have shown TLF could prevent urinary tract infection both in pyelonephritis (PN) rat model and in PN patients. Additionally, our published data demonstrated TLF is helpful to reduce the recurrence of rUTI and protect renal tubular function in clinic. In order to find a novel treating project for rUTI to increase the clinical curative effect, we thus try to combine TLF with CLAT to treat rUTI and design an optimized, pragmatically clinical trial to evaluate the efficacy and safety of this project.

**Methods/design:**

This is a multicenter, double-blind, randomized, controlled clinical trial. We will enroll 200 eligible patients diagnosed with uncomplicated rUTI and then divide them randomly into two groups with a 1:1 ratio: TLF + CLAT group and placebo + CLAT group. This trial consists of two stages, a 12-week period of treatment and a 12-week period of post-treatment follow-up, respectively. The primary outcome will be the recurrence rate at the 12th week of the follow-up period; the second outcomes will be the post-treatment changes in renal and liver function; furthermore, traditional Chinese medicine (TCM) symptoms, non-infection-related physical signs, and subjective symptoms will be scored, and the number of episodes of each subject will be also recorded; meanwhile, vital signs indicators and serious adverse events (SAEs) will be monitored throughout the trial.

**Discussion:**

This study will provide convictive research-derived data to evaluate clinical efficacy and safety of TLF combined with CLAT for rUTI, and provide an evidence-based recommendation for clinicians. Moreover, post-treatment changes in non-infection-related physical signs and subjective symptoms were included in the efficacy evaluation, which is important and more significant for assessing the clinical benefits for those rUTI patients.

**Trial registration:**

Chinese Clinical Trial Registry ChiCTR2100041914. Registered on 10 January 2021. Protocol date and version: September 12, 2020; version 1.

**Supplementary Information:**

The online version contains supplementary material available at 10.1186/s13063-021-05830-4.

## Background

RUTI in adults is defined as repeated urinary tract infection (UTI) with a frequency of 2 or more UTIs in the last 6 months or 3 or more UTIs in the last 12 months [1], which is a common clinical infectious disease, with particular presence in women. It is estimated that 2 in 5 of women will have at least one UTI during their life, and almost one third of them will have recurrence [2]. Antibiotic prophylaxis including continuous low-dose antibiotic therapy is the common treatment for rUTI of the world [3]. However, there are growing concerns about the presumably side effects, adverse reactions, and the increasing risk of bacterial resistance [4]. Given the increasing bacterial resistance, rUTI is becoming refractory more and more [5].

CHM has a recorded history of over 2000 years that may be used in the treatment of rUTI [6]. Previous studies indicated that CHM might be an available treatment method of rUTI for relieving acute UTIs and preventing recurrent episodes [7–9].

TLF is a herbal prescription developed for the treatment of rUTI in the 2000s [10] in the Shanghai Municipal Hospital of Traditional Chinese Medicine. In the past 17 years, our group focuses on the basic and clinical research of rUTI, including in vitro and in vivo, basic and clinical experiments [10–15]. In clinical trials, we find that TLF could effectively reduce the recurrence rate of chronic pyelonephritis (CPN) patients and protect their renal tubular function [10]. In animal experiments, we have built multiple rat models of UTI by Escherichia coli (*E. coli*) O_111_B_4_ successfully, including cystitis, acute pyelonephritis (APN), and CPN rat model, and their results suggested that TLF might effectively inhibit acute and chronic renal tubular and interstitial inflammation caused by *E. coli* retrograde infection, protect renal tubular function, and decrease renal tubular and interstitial fibrosis in rats [11–15].

With the aim to find a novel treating project for rUTI to increase the clinical curative effect, reduce the presumable side effects of CLAT used alone, and improve the clinical benefit for patients, we thus try to combine TLF with CLAT to treat rUTI and design this multicenter, double-blind, randomized, controlled clinical trial to achieve our goal.

## Methods/design

### Primary objective and study hypothesis

This clinical trial aims at obtaining convictive research-derived data to evaluate the efficiency and safety of TLF combined with CLAT for rUTI and improve the clinical benefit for patients. We hypothesize that TLF combined with CLAT will be effective and superior to CLAT alone for increasing the clinical curative effect, reducing the presumably side effects.

### Trial design

**Fig. 1:**
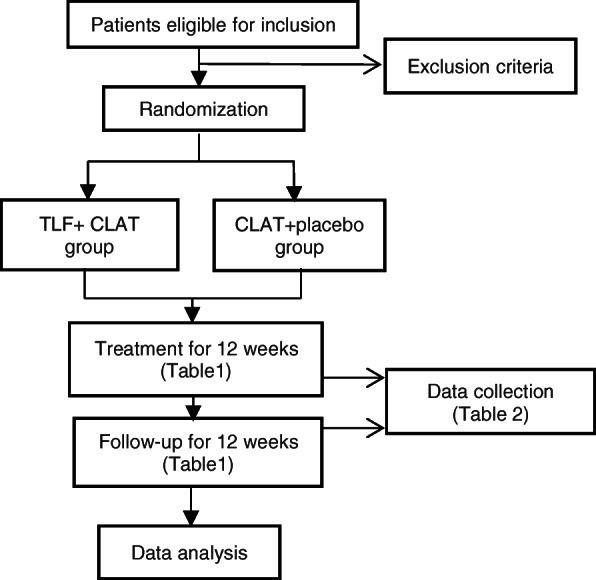
Study flow

We designed a multicenter, double-blind, randomized, controlled clinical trial lasting 24 weeks, including a 12-week treatment period and 12-week follow-up. This trial was designed to comply with the *Declaration of Helsinki* [16] and the *Good Clinical Practice* (GCP) [17] (Fig. 1). The additional file 3 contains the SPIRIT 2013 Checklist.

### Sample size

The sample size will be calculated by PASS (version 15.0) and based on the effective rate of treatment. Group sample sizes of 91 in the treatment group and 91 in the control group achieve 90% power to detect a difference between the group proportions of 0.18. The proportion in the treatment group is assumed to be 0.91, and the proportion in the control group is 0.73 [18]. The significance level of the test is 0.025. With assuming attrition rate of 10%, a planned recruitment target of 200 patients will be set, 100 in each group.

### Screening and recruitment of participants

This study intends to enroll 200 cases mainly from the nephrology outpatient and inpatient departments, including 80 cases from Shanghai Municipal Hospital of Traditional Chinese Medicine, 60 cases from the Sixth People’s Hospital affiliated to Shanghai Jiaotong University, and 60 cases from Baoshan Hospital of Integrative Chinese and Western Medicine Affiliated to Shanghai University of Traditional Chinese Medicine. According to the inclusion and exclusion criteria set based on diagnostic criteria for rUTI and TCM syndrome differentiation, rUTI patients diagnosed with the qi-yin of spleen and kidney deficiency, damp-heat adhesion syndrome by TCM, will be considered as potential participants.

We have taken several methods to ensure recruiting enough participants to reach the target sample size. These methods are shown as follows:

- We have counted the outpatient visits of the Nephrology Departments (sub-centers) of these three hospitals over the past three years in advance. And the statistical results showed that the annual outpatient visits of each Nephrology Department in each hospital exceed 60,000 per year. Therefore, each sub-center has enough rUTI patients to support the progress of this study.
- In order to recruit enough suitable subjects in time, we put recruitment advertisements in all three sub-centers.
- In order to avoid case shedding during observation and follow-up, we have special investigators responsible for contacting the subjects regularly and reminding and helping them to complete the detection and follow-up on time.

### Consent procedures

Investigators in each sub-center will be responsible for performing and obtaining consent before any conduct of the study. All participants will have consented to randomization and trial participation. The consent process will include a clear explanation of the purpose of the trial, possible benefits and risks, alternative treatment options, and subjects' rights and obligations in accordance with the provisions of the Declaration of Helsinki. All participants will have 24 h to decide whether or not to join in. And participants who are willing to be approached will be allocated randomized after signing written informed consent. The informed consent will be held at each test site on a temporary basis, subject to periodic review by the Data Monitoring Committee (DMC), and be archived.

The original version of the informed consent document is provided as a supplemental file. (Additional file 4)

### Study criteria

#### Diagnostic criteria

##### For UTI

According to *Recurrent Uncomplicated Urinary Tract Infections in Women: AUA/CUA/SUFU Guideline* [1] and *Laboratory Diagnosis of Urinary Tract Infections* [19], patients would be diagnosed with UTI if:

1. ≥10^5^colony-forming units (cfu)/ml bacteria in a clean-catch specimen (urine that had already stay in the bladder for more than 4–6 h);
2. White blood cell (WBC)>10/HP in urinary sediment, or with clinically symptoms of UTI; UTI can be diagnosed according to (1) and (2). If without (2), UTI diagnosis should not be confirmed until the quantitative urine culture rechecked is still ≥10^5^cfu/ml, and the bacteria are the same strain as the former test. Or can be diagnosed if the laboratory test show one of the following:
3. Culture positive for bacteria in the examination of urine in the bladder (bacteria of any quantity is considered);
4. Gram stain of a centrifuged urine specimen can be used for urine sample which is unavailable to do quantitative urine culture. With clinical symptoms, diagnosis can be confirmed if the bacteria more than 1 per oil immersion field; and
5. Urine sample should be reexamined if its colony count≥10^4^cfu/ml but<10^5^cfu/ml, and UTI diagnosis ought to be based on clinical symptoms or examination of urine in the bladder if its colony count does not change.

##### For rUTI

UTI is considered recurrent if with more than 2 episodes in the last 6 months or more than 3 episodes in the last 12 months [1].

#### TCM syndrome differentiation

rUTI Patients will be diagnosed with the qi-yin of spleen and kidney deficiency and damp-heat adhesion syndrome by TCM if they have at least one primary symptom and more than two secondary symptoms or two primary symptoms listed below [20].

#### Primary signs and symptoms

- Repeated episodes of dysuria, frequency, and urgency, particularly more severe after fatigue
- Weakness or pain in the waist and knees

#### Secondary signs and symptoms

- Feverish palms and soles
- Xerocheilia
- Spontaneous or night sweating
- Red tongue with less coating, thready and fast or deep and weak pulse

#### Inclusion/exclusion criteria

Patients will be eligible for the trial if they meet all of the following criteria:

- In line with diagnostic criteria for rUTI stated above
- Meet the diagnostic criteria of TCM syndrome differentiation stated above
- Age 18 to 70 years
- Agree to take part in the trial and sign an informed consent

Patients will be ineligible if they have any of the following criteria:

- New-onset acute urinary tract infection
- In-dwelling catheter
- Known structural or functional urological abnormalities, such as urinary tract congenital malformation, renal pelvis calculi, or other lesions
- Urethral syndrome
- Chronic kidney disease (CKD) stage IV-V (eGFR<30 ml/min)
- Combine with serious heart and liver function damage or diabetes and other diseases which need immediate treatment
- Allergic to two or more kinds of antibiotics of this study
- Pregnancy or lactation (women of childbearing age is demanded to have a urine HCG test)
- Severe central nervous system disease
- Be participating in other drug clinical trials or have participated in other clinical trials in the last 3 months

#### Participant withdrawal criteria

Participant will withdraw or be rejected for further participation if:

- Voluntarily quitting
- Poor adherence
- Any violation of the inclusion criteria
- Allergic to two or more of the originally intended drugs or have other specific and intolerable adverse reactions
- Drop-out or loss of follow-up during observation
- Later examination finds other serious diseases (serious heart and liver function damage, blood disease, etc.) or special physiological changes (pregnancy, etc.) and not fit to continue the study

The reasons for the excluded cases will be explained, and the case report form will be kept for future reference. Anyone withdrawn within two weeks after receiving treatment will not be included in the per-protocol population and analyzed as missing data.

#### Trial suspension criteria

Trial will be suspended if serious adverse events (SAEs) occurred during the trial.

##### Adverse events (AEs)

Our group has engaged in the basic and clinical researches of TLF against rUTI since 2000s, and our published data demonstrated TLF is helpful to reduce the recurrence of rUTI and protect renal tubular function in the clinic [10–15]. Therefore, we are very confident that the patients treated with TLF will not have AEs. It is reported that CLAT has potential risks of pulmonary, renal, and hepatic toxicity, and may cause gastrointestinal disturbances and skin rash [1].

All adverse events (AEs) will be collected systematically by asking participants specifically every 2 weeks and be recorded in detail on the specific form throughout the study. All relevant AEs will be reported in trial publications in the end.

##### Serious adverse events (SAEs)

Events that could lead to any of the following outcomes are included in the serious adverse events (SAEs), which will be observed, monitored, and judged by researchers, and gave timely treatment and reported to relevant authorities.

- Hospitalization or prolonged treatment
- Cripple or life-threatening complications
- Fetal deformity or significant disability or death

### Randomization

After signing written informed consent and completing baseline data collection, patients eligible for the trial in each trial site will be assigned randomized by investigators with 1:1 ratio to the simple random method, using a computer-generated random number sequence conducted by an independent statistician using SPSS (version 21.0). Sealed, opaque envelopes with printed randomization numbers on them are available in each center. According to the sequence of patients entering clinical observation, the corresponding random number was selected.

Following allocation, the subjects will receive corresponding treatment and be ensured continued supply for 12 weeks by a clinician in each site.

### Blinding

This trial follows the double-blind design. The division of the groups in this study will be blinded to all participants, investigators, and statisticians. The placebo and TLF are the same in the appearance of the drug package. What’s more, the drug distribution will be conducted by a pharmacist who is not involved in the study.

To assess whether participants stayed masked or knew their assignment, we will use the direct questioning method at the end of follow-up, that is, design a question in the CRF to ask the subjects to guess which group they are assigned to and ask them to give the basis or reason of guess. Then calculate blinding index (BI) according to the guess result and the implementation of the blind method can be judged.

### Intervention

Patients who are diagnosed with non-acute uncomplicated rUTI or were in remission after 14 days of treatment with sensitive antibiotics during acute episodes will be enrolled after signing informed consent. Then they will be randomly assigned to the treatment group or the control group.

### Treatment group

Participants randomly assigned to the treatment group will be administered TLF combined with CLAT.

#### Dosage and duration (Table 1)

TLF: one package as an oral dose four times daily (15 min after three meals and 1 h before bedtime) for 12 weeks;

**Table 1:** Interventions: administration methods and duration in the experimental and control group

Treatment	Time					
	Week 0–2	Week 3–4	Week 5–6	Week 7–8	Week 9–10	Week 11–12
**TLF**	**○**	**○**	**○**	**○**	**○**	**○**
**Placebo**	**●**	**●**	**●**	**●**	**●**	**●**
**CLAT**						
**Levofloxacin** (0.1 g, QN, po)	**○●**			**○●**		
**Nitrofurantoin** (0.1 g, QN, po)		**○●**			**○●**	
**Cefdinir** (0.1 g, QN, po)			**○●**			**○●**

A white circle represents the treatment of the treatment group; a black circle represents the treatment of the control group

CLAT: first, levofloxacin 0.1 g taken orally once per night for 2 weeks; next, nitrofurantoin 0.1 g taken orally once per night for 2 weeks; then, cefdinir 0.1 g taken orally once per night for 2 weeks. And then repeat one turn in accordance with the medication order above, the total course of antimicrobial therapy is 12 weeks. All antibiotics are taken 15 min after dinner. For those allergic to any of the three antibiotics, trimethoprim-sulfamethoxazole (TMP 40 mg, SMZ 200 mg) one tablet per day orally or fosfomycin-trometamol (3 g, qod, po) will be used as the alternative drug.

### Control group

Participants randomly assigned to the control group will receive placebo plus CLAT the dosage and administration of which are the same as the treatment group (Table 1).

We will conduct health education for all groups. And the method of drug administration will be instructed to all co-investigators and patients. The TLF granules will be manufactured by Jiangyin Tianjiang Pharmaceutical Co. LTD.

#### Treatment of acute-onset UTI during the treatment period

Conduct urine culture, antimicrobial susceptibility testing, and the colony count immediately, then give a sufficient amount of oral or parenteral antibiotics for 7–14 days. Acute-onset infections should be treated with culture-directed antibiotics according to patients’ existing antimicrobial susceptibility testing records at first. In other situations, antibiotics will be prescribed empirically while awaiting the result of urine culture.

Such cases mentioned above should be marked as no recovery (NR).

#### Concomitant medications

Non-antibiotic drugs or products that may affect efficacy judgments are specifically prohibited during this trial, including cranberries, lactobacillus, D-mannose, methenamine, and other herbs. Any drugs other than those used to treat rUTI must be recorded on the observation sheet, including drug name, dose, and treatment duration.

### Items to be measured and the time points of data collection

Patient data, efficacy-related examinations, and indicators will be performed and collected at 2 weeks before baseline, baseline, and every 2 weeks or 4 weeks during treatment and follow-up period.

Measurement items and the time points of data collection are listed in Table 2.

**Table 2:**
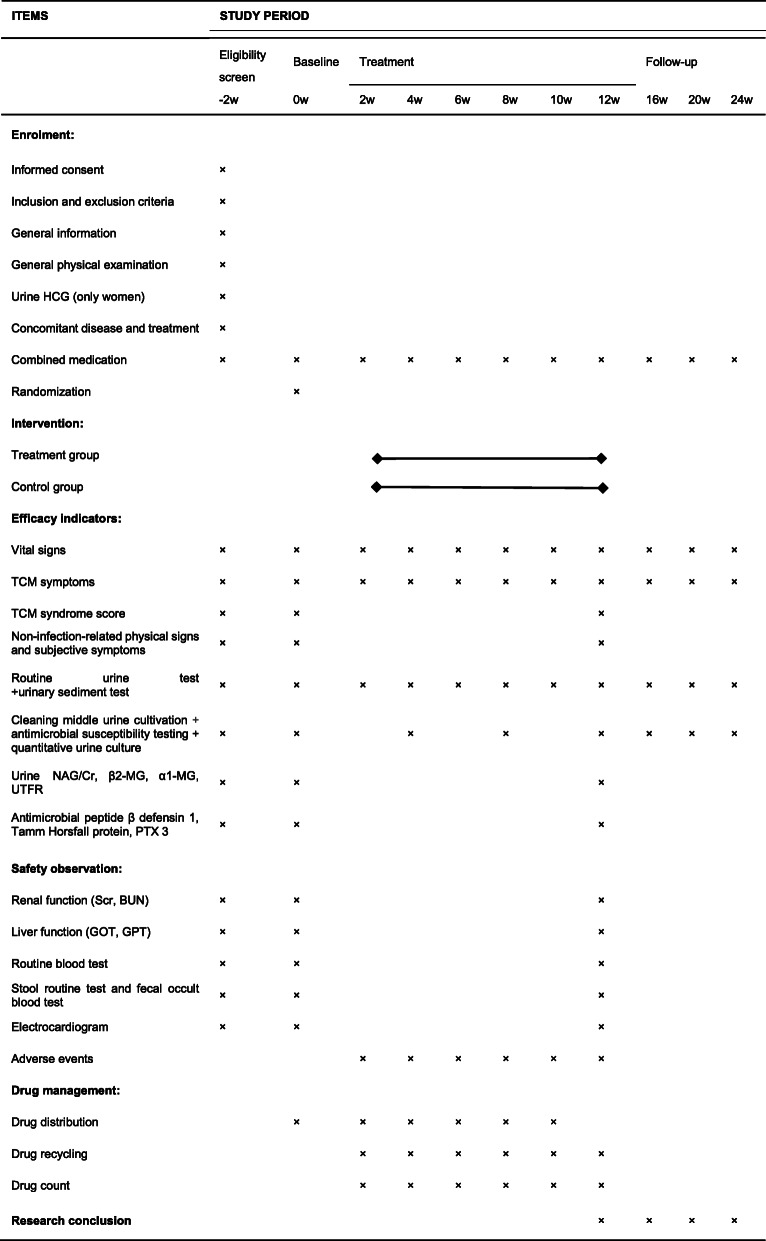
SPIRIT figure: Items to be measured and time points of data collection

*HCG* human chorionic gonadotropin, *TCM* traditional Chinese medicine, *Urine NAG/Cr* urine glucosaminidase/creatinine, *β2-MG* β2-microglobulin, *α1-MG* α1-microglobulin, *UTFR* urinary transferrin, *PTX 3* pentraxin 3, *Scr* serum creatinine, *BUN* blood urea nitrogen, *GOT* glutamic oxalacetic transaminase, *GPT* glutamic-pyruvic transaminase

Blood and urine samples will be collected at each site and transported to the central laboratory for unified measurement, analysis, and eventual destruction. No additional collection of samples will be obtained for storage or future testing during the conduct of this trial. All samples will be coded with their unique identifier and logged in the electronic data systems (EDS). The researchers who will carry out analyses on these samples will not have access to any information of subjects or intervention.

The following methods should be used to minimize attrition and reduce potential loss to follow-up in this clinical trial.

- Detailed introduction for patients including the whole process of the clinical study and its requirements and possible risks
- Special investigators will be responsible for contacting the subjects by telephone regularly and helping subjects receive treatment and testing on time

## Outcomes

### Primary efficacy outcome

The primary efficacy outcome will be the recurrence rate at the 12th week of the follow-up period.

### Secondary efficacy outcomes

The secondary efficacy outcomes will be the post-treatment changes in renal and liver function, including serum creatinine (Scr), blood urea nitrogen (BUN), glutamic oxalacetic transaminase (GOT), and glutamic-pyruvic transaminase (GPT). Furthermore, TCM symptoms, non-infection-related physical signs, and subjective symptoms will be scored, and the number of episodes of each subject will be recorded. Meanwhile, vital signs indicators and SAEs will be monitored throughout the trial.

### Safety indicators

- Incidence of SAEs
- Vital signs indicators (electrocardiogram, blood routine, urine routine, fecal routine and occult blood test, liver and renal function test)

## Therapeutic effect evaluation

### TCM syndrome scores

According to the *Guidelines for Clinical Research of Chinese Medicine (New Drug)* [21], the primary symptoms of the qi-yin of spleen and kidney deficiency and damp-heat adhesion syndrome will be scored 2, 4, and 8, while secondary symptoms will be scored 1, 2, and 4. The scores will be added for each patient, separately. The evaluation was conducted once before treatment and the 12th week after.

The two total scores calculated by the measurement scale for TCM symptoms before treatment and the 12th week after for each patient will be added together, and then used to calculate an efficacy indicator (EI) for the evaluation of treatment efficacy:

EI = (Total symptom score at baseline − Total symptom score post-treatment)/Total symptom score at baseline × 100%

Symptom improvement degree will be presented in four levels showed in Table 3.

**Table 3:** The degree of symptom improvement

Degree	Indicators			
	EI	Clinical symptoms and signs	Urine routine (for two consecutive times)	Clean-catch midstream urine culture
Full recovery (FR)	≥ 95%	Disappeared	Normal	Negative
Good recovery (GR)	<95% ≥70%	Disappeared or nearly disappeared	Normal or almost normal	Negative
Modest recovery (MR)	<70% ≥30%	Alleviated	Significantly improved	Occasionally positive
No recovery (NR)	<30%	No significant improvement	No significant improvement	Colony count ≥10^5^/ml 4 weeks after medication

### Relapse or reinfection

With symptoms of urinary irritation suddenly aggravated, and quantitative culture for bacteria in clean-catch midstream urine shows bacterial colonies ≥ 10^5^cfu/ml, that may be due to relapse (with the same strain of organism) or reinfection (with a different strain or species of organism) [1]. Importantly, it is necessary to exclude specimen contamination and gynecological inflammation.

### Statistics of recurrence

Recurrence rate of UTI of each patient during the treatment until the end of the follow-up will be calculated separately and compared at the end of the trial.

### Semi-quantitative assessment of non-infection-related physical signs and subjective symptoms

Non-infection-related physical signs and subjective symptoms include dysuria and urgency, underbelly distension, nocturia, low back pain or weakness, self-diagnosed UTI, and costovertebral angle tenderness [22]. Such symptoms and signs will be scored according to a semi-quantitative integral method, namely, classified into non-symptoms, mild symptoms, moderate symptoms, and severe symptoms, and represented by −, +, ++, and +++, with 0, 1, 2, and 4 points, correspondingly. The evaluation will be conducted before treatment and the 12th week after separately.

### Short-term and long-term cure rate

It will be recorded as a short-term cure if present of *full recovery* (FR) 4 weeks after the end of the medication and be recorded as a long-term cure if present of FR 12 weeks after the end of the medication [21] (Table 3).

### Safety evaluation

Safety evaluation will be performed at the time of the beginning and the end of the trial, including (1) blood routine, stool routine, and stool occult blood; (2) liver and kidney function; and (3) electrocardiogram. Keep in touch with the participants to keep abreast of adverse events or emergencies. The relationship between the experimental drugs and adverse events will be assessed with five grades of “positively related, possibly related, impossible to judge, possibly unrelated, and definitely unrelated” [21], so as to judge the adverse reactions and to count the incidence of it.

### Post-trial care and compensation

We will take necessary measures to ensure the safety of the subjects during the trial. Each subject will be reimbursed for transportation expenses. Our study center will provide insurance for non-negligent harm associated with this trial. In addition, incidences judged to arise from negligence harm (including those due to major protocol violations) will not be covered by the study insurance policies.

### Management of test drugs

The test drugs used during the trial will be specially managed by qualified pharmacists in each trial center, and the experimental drugs that need special preservation (such as light avoidance, dry, etc.) will be kept according to the requirements of the protocol. The test drugs will not available to any non-clinical trial participants and are intended only for patients enrolled for this clinical trial. Dose and usage are in accordance with the protocol strictly. The remaining test drugs will be returned or destroyed as required. Record the time, quantity, recipient, and so on of the handing out and reclaiming of the test drugs and strictly follow the record and modification specifications.

## Data management and analysis

### The roles and responsibilities of committees during this study

Investigators will be responsible for performing and obtaining the consent before any conduct of the study, recruitment, and allocation of participants to their randomized group, collection of data, and completion of the case report forms (CRFs), along with follow-up of study patients in each site. The study conduct and data collection will be monitored by DMC and SC (Table 4) to ensure that the study is conducted as GCP required. All monitoring findings will be reported in a timely manner.

**Table 4:** The roles and responsibilities of committees during this study

Committees	Composition	Roles and responsibilities
Data Monitoring Committee (DMC)	Principle investigator, research physician, administrator, statistician	• Understand the protocol of the study • Have clinical experience and relevant professional knowledge • Organize an initiating meeting, any other investigator meeting, and site visiting • Budget administration and contractual issues with individual centers • Randomization • Audit feedback forms and verify data • Advice for lead investigators • Organize central sample collection, analysis, and eventual destruction • Responsible for trial master file • Provide risk report to regulatory agency and ethics committee
Steering Committee (SC)	Monitor, lead investigator, coordinator	• Agreement of final protocol • Organize investigator training • Monitor enrolment, allocation, collection of data, and completion of CRFs • Auditing study conduct and data collected in each site • Reviewing the progress of the study and if necessary agreeing changes to the protocol and/or investigators brochure to facilitate the smooth running of the study

### Data entry and management

Data entry and administration are the responsibility of designated data administrators by the data management system (DMS). Manual double entry of data will be used to avoid the mistake of data entry. Data collected during the course of the research will be kept strictly confidential and only data managers and statisticians have access to the data until the end of the trial. Data administrators, statisticians, and researchers will be kept blinded from treatment allocation. With the provisions of GCP, data will be kept for 5 years after the trial completes.

### Data analysis

Statistical analysis data set includes intention-to-treat (ITT) and per-protocol population. ITT will be applied to the full analysis set (FAS) consisting of all randomized subjects. Subjects who have received two or more weeks of treatment will be included in the per-protocol population. Both two sets will be analyzed at the end of this study. The reasons for excluding patients from the Per-Protocol Population should be recorded clear and documented prior to breaking blindness. Statistical analysis results of the two data set will be compared and analyzed after the trial completes.

Statistical analysis includes (1) baseline data analysis, (2) effectiveness analysis, and (3) security analysis. SPSS21.0 statistical analysis software will be used for statistical analysis. Two-sided test will be used for all statistical tests, and a *P* value less than 0.05 was considered statistically significant. Multiple imputation will be applied to missing data, and sensitivity analysis will be conducted to demonstrate the robustness of the results, when the high missing data proportions occur.

### Quality control of data

We will strictly control the data quality from the following aspects:

- Before the start of the trial, the experiment scheme was designed on the foundation of professional background knowledge and clinical experience of all participants and researchers, so as to ensure the scheme is scientific and operable.
- Patients will be selected in strict compliance with inclusion and exclusion criteria.
- The randomized, double-blind design will be followed throughout the experiment.
- Strictly follow the experimental protocol for treatment, testing, and evaluation of efficacy.
- Researchers, cases observers, investigators, and data entry personnel will be trained regularly to ensure that any observation and examination results in the test are recorded in the CRF in a true, accurate, complete, and timely manner. Each site will be visited termly by the trial monitor.
- The data in CRF will come from and be consistent with the original file. Alteration is forbidden to be made at will. Any correction shall be made by keeping the original record legible and signed by the person making the correction.
- Ensure the subject’s safety and record timely. If SAE occurs during the clinical trial, take appropriate treatment for the subject and report to the responsible person immediately.
- Statistical experts are involved in designing experiments and evaluating data until the end of the study.
- DMC is independent from the funder and has no competing interests with the study organizers.

### Confidentiality

All paper files containing names or other personal identifiers on all subjects participating in the study, such as informed consent forms, will be stored in a safe with limited access. Our database has been set with strict access restrictions and be secured with password-protected access systems. All study records, including reports, point-of-care test specimens will be identified by an identification number to maintain participant confidentiality. With the provisions of the Good Clinical Practice (GCP), data will be kept for 5 years after the trial completes.

### Unblinding

Unblinding procedures are divided into two parts. The first unblinding will be performed after the data collection was complete. In this step, all data will be distinguished into two groups, without specifying which is the control or treatment group. After the statistical analysis is completed, the second unblinding will be conducted to uncover which is the control group or the treatment group. Emergency unblinding is permissible if necessary for safety reasons, such as SAEs that occurred during the trial. The reason and time of emergency unblinding will be documented and reported.

### Ethics approval

This study must be conducted in accordance with the Declaration of Helsinki [16]. Before the experiment, the Ethics Committee shall conduct an ethical review on the research plan and carry out the research after it is approved (more detailed information about the ethical approval is listed in Additional file 1).

Any important protocol modifications (e.g., changes to study design, sample sizes) which may impact on the conduct of the study will be reported and agreed upon by the relevant parties (e.g., funders, trial registries, and journals), and approved by the Ethics Committee prior to implementation.

### Dissemination

Future trial presentation or publication will be circulated to the principal investigators of the center study site. All participants who contribute substantive to the design, conduct, interpretation, and reporting of the trial are recognized through the granting of authorship on the final trial report. Professional authors are not needed.

## Discussion

Nowadays, AMR is becoming a serious threat to public health, and the risk of it is getting higher and higher, which means antibiotics may be less effective and couldn’t meet clinical needs in the future [4, 5]. Facing such a situation, more rigorous clinical studies are needed to test the efficiency and safety of non-antibiotics methods against rUTI, such as CHM [7, 23].

TLF is a herbal prescription that consists of Daxueteng (*Sargentodoxa cuneata*), Huzhang (*Polygonum cuspidatum*), Shengmiren (Raw Coix Seed), etc. [10]. *E. coli* is the most common causative organism of UTI both in North America and China [1, 24]. Current pharmacology researches show that chlorogenic acid, which is the effective component of Sargentodoxa Cuneata could inhibit the activities of *E. coli* and *Streptococcus faecalis* [25]. 3,5-dimethoxy-1H-inden-1-one extracted from Coix Seed shows a wide antimicrobial activity against bacteria [26]. The extract of *Polygonum cuspidatum* has an inhibitory effect on *E. coli* and *Pseudomonas aeruginosa* [27].

Our group has engaged in the basic and clinical research of rUTI for more than 17 years. We have built multiple rat models of UTI by *E. coli* O_111_B_4_ successfully, including cystitis, APN, and CPN rat models, and conducted extensive clinical research on this herbal prescription [10–15]. Our previously published data have shown clearly that TLF might effectively reduce the recurrence rate of rUTI and protect the renal tubular function, which suggests that TLF might be a potential effective prescription for rUTI.

Due to the increasing AMR, more potential side effects, and lower clinical efficacy, rUTI is becoming refractory more and more [5]. Obviously, a rational, pragmatically, more effective and safer treatment is urgently needed to improve this unsatisfactory situation. Therefore, we try to combine TLF with CLAT to treat rUTI and design this multi-center, double-blind, randomized, controlled clinical trial to evaluate the efficiency and safety of this novel treatment.

Based on clinical experience and previous studies [10–15], we hypothesize that this treatment will decrease the recurrence rate of rUTIs, reduce the adverse reactions and side effects of CLAT alone, and protect the renal tubular function of rUTI patients.

Studies [4] have indicated that rUTI has considerable impacts on various aspects of the patients, including social and economic burden, health-related quality of life, and so on. We sincerely hope that the successful implementation of this study would make a significant contribution to the prevention and treatment of rUTI. This therapy might be a promising way to reduce the economic burden of rUTI patients, since it might help to reduce the usage of antibiotics as well as the recurrence rate of rUTI. In addition, rUTI is a clinical refractory disease with considerable morbidity. It is estimated that 2 in 5 of women will have at least one UTI during their life, and almost one third of them will have recurrence [2]. Symptoms such as abdominal distention and fatigue would persist for a considerable time even after the infection is under control, and would induce restricted activity, work absenteeism, and longer time spent confined to bed. This new therapy protocol hopefully would help to improve the health-related quality of life of rUTI patients by alleviating the non-infection-related physical signs and subjective symptom and to reduce the presumably side effects of antibiotic therapy alone.

## Trial status

This trial was registered at the Chinese Clinical Trial Registry (registration number: ChiCTR2100041914) in January 2021. Recruitment will begin in August 2021 and it is anticipated that enrollment will be completed in December 2023.

## Supplementary Information

**Additional file 1.** Ethical approval document: Application Form for Ethical Review of Clinical Research Projects.

**Additional file 2.** SPIRIT 2013 Checklist: Recommended items to address in a clinical trial protocol and related documents.

**Additional file 3.** Funding documentation.

**Additional file 4.** The informed consent document.

## Data Availability

The datasets used and/or analyzed during the current study are available from the corresponding author on reasonable request.

## Abbreviations

rUTI: Recurrent urinary tract infection
UTI: Urinary tract infection
TCM: Traditional Chinese medicine
TLF: Tailin formulation
CLAT: Continuous low-dose antibiotic therapy
AES: Adverse events
EI: Efficacy indicator
HCG: Human chorionic gonadotropin
Urine NAG/Cr: Urine glucosaminidase/creatinine
β2-MG: β2-microglobulin
α1-MG: α1-microglobulin
UTFR: Urinary transferrin
THP: Tamm-Horsfall protein
PTX 3: Pentraxin 3
Scr: Serum creatinine
BUN: Blood urea nitrogen
GOT: Glutamic oxalacetic transaminase
GPT: Glutamic-pyruvic transaminase
Po: Per os
QN: Once per night
QOD: Once every other day
QID: Four times a day
FR: Full recovery
GR: Good recovery
MR: Modest recovery
NR: No recovery
ITT: Intention to treat principle
CRF: Case report form
