# Supplementary material for: Clinical efficacy of Tailin formulation combined with continuous low-dose antimicrobial therapy for recurrent urinary tract infection: study protocol for a multicenter, double-blind, randomized, controlled clinical trial
Source: Trials. 2021 Dec 11;22:901. doi: 10.1186/s13063-021-05830-4 (PMC8665585; doi:10.1186/s13063-021-05830-4)
Supplement: Supplementary file 1 — Additional file 1. Ethical approval document: Application Form for Ethical Review of Clinical Research Projects. [file 13063_2021_5830_MOESM1_ESM.pdf]

上海市中医医院伦理委员会
临床科研项目伦理审查批件

批件号：2020SHL-KY-47

项目名称	泰淋方联合抑菌疗法中西医结合方案治疗复发性尿路感染随机、对照、多中心临床研究		
任务来源	上海市教委高峰高原学科建设	课题编号	
研究负责单位	上海市中医医院		
本中心课题责任人（姓名及职称）	龚学忠 主任医师		
审查日期	2020 年 10 月 29 日	审查地点	上海市芷江中路 274 号
审查类别	初始审查	审查方式	会议审查
审查委员	朱凌云、李雁、任建琳、樊天佑、张立超、张洁、孙冉冉		
审查文件	1、伦理审查申请表（申请者签名并注明日期）<br>2、项目任务书<br>3、方案<br>4、知情同意书<br>5、病例报告表<br>6、研究组人员名单		
审查意见	根据国家卫计委《涉及人的生物医学研究伦理审查办法》（2016），国家食品药品监督管理总局《药物临床试验质量管理规范》(2003)、《医疗器械临床试验质量管理规范》（2016），WMA《赫尔辛基宣言》（2013）、CIOMS《人体生物医学研究国际道德指南》（2002），《药物临床试验伦理审查工作指导原则》（2010），《中医药临床研究伦理审查管理规范》(2010)，经本伦理委员会审查，意见如下：<br>请遵循保护受试者的健康和权利的原则。进行方案、知情同意书等修改时要通知伦理委员会，并按照伦理委员会批准的最新版本的方案实施研究，使用伦理委员会批准的最新版本、有效的知情同意书纳入受试者；及时报告与研究有关的严重的和意外的不良事件；及时报告无法预料的情况、暂停/终止研究、或其他伦理委员会的重要决定；随时应伦理委员会的要求，报告正在进行的研究的有关信息；试验结束，请提交结题报告。		
审查结果	■ 同意<br>□ 必要的修改后同意	□ 不同意<br>□ 终止或暂停已同意的研究	
年度/定期跟踪审查频率	请于 2021 年 11 月 3 日前 1 个月递交研究进展报告		
有 效 期	2020 年 11 月 4 日至 2021 年 11 月 3 日		
联系人与联系电话	凌丽 021-56628310		
主任委员签字	[illegible]		
盖 章	上海市中医医院伦理委员会		
日 期	2020年10月31日		

Application Form
for Ethical Review of Clinical Research Projects

Identifier: 2020SHL-KY-47

Project Name	A multi-center, double-blind, randomized, controlled clinical trial for the treatment of recurrent urinary tract infection by Tailin formulation combined with continuous low-dose antibiotic prophylaxis project		
Project Sources	Shanghai Municipal Education Commission Gaofeng Gaoyuan Discipline Construction Project	Identifier	No.02.ZY05.191311N
Research Institution in Charge	Shanghai Municipal Hospital of Traditional Chinese Medicine		
Responsible Person	Xuezhong Gong, Chief Physician		
Date of Review Beginning	October 29, 2020	Review Site	No. 274, Zhijiang Middle Road, Shanghai
Review Categories	Initial review	Review Method	Committee meeting
Committee Members	Lingyun Zhu, Yan Li, Jianlin Ren, Tianyou Fan, Lichao Zhang, Jie Zhang, Ranran Sun		
Application Documents	1. Application form for ethical review (signed by applicant and dated)<br>2. Project charter<br>3. Study protocol<br>4. Informed consent form<br>5. Case report form (CRF)<br>6. List of research members		
Review Comment	According to the *Methods for the Ethical Review of Biomedical Research Involving Human Subjects (2016)* issued by the National Health and Family Planning Commission, *the Good Clinical Practice (2003)*, *Practice for Quality Management of Clinical Trials of Medical Devices(2016)* issued by China Food and Drug Administration, *Declaration of Helsinki(2013)* issued by World Medical Association(WMA), *International Ethical Guidelines for Biomedical Research Involving Human Subjects(2002)* issued by Council For International Organization Of Medical Sciences(CIOMS), *Guiding Principles for Ethical Review of Drug Clinical Trials(2010)*, *Practice of Ethics Review and Management for Clinical Research of Traditional Chinese Medicine(2010)*, and upon review by the Ethics Committee, the opinions are as follows:<br>Please follow the principles of protecting the health and rights of the subjects. Inform the Ethics Committee of any changes of the study protocol, informed consent, etc. The study should be carried out according to the latest approved version of the protocol, and recruit subjects using the latest version of valid informed consent form approved by the Ethics Committee. Report serious and unexpected adverse events related to the study, unforeseen circumstances, suspension/termination of the research, or other important decisions of the Ethics		

	Committee in a timely manner. Report relevant information on ongoing research whenever the Ethics Committee request. Submit the conclusive report after the study completed.
Review Result	■ Agree<br>□ Disagree<br>□ Agree after necessary modifications<br>□ Terminate or suspend the agreed studies
Frequency of Annual/Periodic Supervision Reviews	The study progress report should be submitted one month before November 3, 2021
Validity	From November 4, 2020 to November 3, 2021
Contact	Li Ling　Tel:021-56028310
Signature of Chairman	Lingyun Zhu
Seal	
Date of Review Completion	October 31, 2020
