# Supplementary material for: Clinical efficacy of Tailin formulation combined with continuous low-dose antimicrobial therapy for recurrent urinary tract infection: study protocol for a multicenter, double-blind, randomized, controlled clinical trial
Source: Trials. 2021 Dec 11;22:901. doi: 10.1186/s13063-021-05830-4 (PMC8665585; doi:10.1186/s13063-021-05830-4)
Supplement: Supplementary file 3 — Additional file 3. Funding documentation. [file 13063_2021_5830_MOESM3_ESM.pdf]

关于国家自然科学基金资助项目批准及有关事项的通知

龚学忠　先生/女士：

根据《国家自然科学基金条例》的规定和专家评审意见，国家自然科学基金委员会（以下简称自然科学基金委）决定批准资助您的申请项目。项目批准号：81873280，项目名称：从BNIP3和PINK1/Parkin介导的线粒体自噬和线粒体质量控制研究对比剂急性肾损伤的发病机制及“制大黄-川芎”药对的肾保护机制，直接费用：62.00万元，项目起止年月：2019年01月至 2022年 12月，有关项目的评审意见及修改意见附后。

请尽早登录科学基金网络信息系统（https://isisn.nsfc.gov.cn），获取《国家自然科学基金资助项目计划书》（以下简称计划书）并按要求填写。对于有修改意见的项目，请按修改意见及时调整计划书相关内容；如对修改意见有异议，须在计划书电子版报送截止日期前提出。

计划书电子版通过科学基金网络信息系统（https://isisn.nsfc.gov.cn）上传，由依托单位审核后提交至自然科学基金委进行审核。审核未通过者，返回修改后再行提交；审核通过者，打印为计划书纸质版（一式两份，双面打印），由依托单位审核并加盖单位公章后报送至自然科学基金委项目材料接收工作组。计划书电子版和纸质版内容应当保证一致。向自然科学基金委提交和报送计划书截止时间节点如下：

1、提交计划书电子版截止时间为**2018年9月11日16点**（视为计划书正式提交时间）；

2、提交计划书电子修改版截止时间为**2018年9月18日16点**；

3、报送计划书纸质版截止时间为**2018年9月26日16点**。

请按照以上规定及时提交计划书电子版，并报送计划书纸质版，未说明理由且逾期不报计划书者，视为自动放弃接受资助。

附件：项目评审意见及修改意见表

国家自然科学基金委员会
医学科学部
2018年8月16日

国家自然科学基金资助项目批准通知

龚学忠　先生/女士：

根据《国家自然科学基金条例》规定和专家评审意见，国家自然科学基金委员会（以下简称自然科学基金委）决定资助您申请的项目。项目批准号：82074387，项目名称：从GPX4、p62/Nrf2/HO-1介导的肾小管上皮细胞铁死亡研究三价砷致肾损伤的发病机制及川黄方的肾保护机制，直接费用：55.00万元，项目起止年月：2021年01月至 2024年 12月，有关项目的评审意见及修改意见附后。

请尽早登录科学基金网络信息系统（https://isisn.nsfc.gov.cn），获取《国家自然科学基金资助项目计划书》（以下简称计划书）并按要求填写。对于有修改意见的项目，请按修改意见及时调整计划书相关内容；如对修改意见有异议，须在电子版计划书报送截止日期前向相关科学处提出。

电子版计划书通过科学基金网络信息系统（https://isisn.nsfc.gov.cn）上传，依托单位审核后提交至自然科学基金委进行审核。审核未通过者，返回修改后再行提交；审核通过者，打印纸质版计划书（一式两份，双面打印），依托单位审核并加盖单位公章，将申请书纸质签字盖章页订在其中一份计划书之后，一并将上述材料报送至自然科学基金委项目材料接收工作组。电子版和纸质版计划书内容应当保证一致。**自然科学基金委将对申请书纸质签字盖章页进行审核，对存在问题的，允许依托单位进行一次修改或补齐。**

向自然科学基金委补交申请书纸质签字盖章页、提交和报送计划书截止时间节点如下：

1. **2020年10月23日16点：** 提交电子版计划书的截止时间（视为计划书正式提交时间）；

2. **2020年10月30日16点：** 提交电子修改版计划书的截止时间；

3. **2020年11月06日16点：** 报送纸质版计划书（其中一份包含申请书纸质签字盖章页）的截止时间。

4. **2020年11月27日16点：** 报送修改后的申请书纸质签字盖章页的截止时间。

请按照以上规定及时提交电子版计划书，并报送纸质版计划书和申请书纸质签字盖章页，未说明理由且逾期不报计划书或申请书纸质签字盖章页者，视为自动放弃接受资助；未按要求修改或逾期提交申请书纸质签字盖章页者，将视情况给予暂缓拨付经费等处理。

附件：项目评审意见及修改意见表

国家自然科学基金委员会

2020年9月27日

中医学团队年度研究经费下拨方案

为保障中医学学科建设任务的顺利开展和建设目标的及时完成，我学科对中医学顶尖优势团队（8 支团队）和中医学高峰高原创新团队（共 19 支团队）下拨年度研究经费，并请相关团队按照要求，以目标为导向，成果为产出，合理按时使用经费。具体下拨经费金额见附表。现将名单报备科技处（学科建设办公室），并请财务处将校内团队另立卡，谢谢！

中医学学科

2020 年 7 月 1 日

中医学高峰高原创新团队名单：

序号	团队名称	序号	团队名称
1	刘　平顶尖优势团队	15	刘　胜高峰高原团队
2	杨华元顶尖优势团队	16	曹永清高峰高原团队
3	季　光顶尖优势团队	17	曹月龙高峰高原团队
4	杨永清顶尖优势团队	18	高月求高峰高原团队
5	房　敏顶尖优势团队	19	刘成海高峰高原团队
6	王拥军顶尖优势团队	20	胡义扬高峰高原团队
7	李　琦顶尖优势团队	21	杨　巍高峰高原团队
8	吴焕淦顶尖优势团队	22	张慧敏高峰高原团队
9	沈雪勇高峰高原团队	23	许　玲高峰高原团队
10	王忆勤高峰高原团队	24	李　斌高峰高原团队
11	陈　晓高峰高原团队	25	王振宜高峰高原团队
12	龚学忠高峰高原团队	26	朱文伟高峰高原团队
13	邓跃毅高峰高原团队	27	许家佗高峰高原团队
14	阙华发高峰高原团队		

Annual plan of research fund allocation for the Traditional Chinese Medicine team

To support the construction of Traditional Chinese Medicine (TCM) discipline, we provide annual research funding for the Top Team of TCM (8 in total), and the Gaofeng Gaoyuan Innovation Team of TCM (19 in total). Relevant teams are requested to follow the requirements, be target-oriented, outcome-oriented, and use funds reasonably and on time. The specific amount of appropriated funds is shown in the attached table. Please submit the list to the Science and Technology Department (Discipline Construction Office), and ask the Finance Department to register the campus team separately. Thank you!

Traditional Chinese Medicine (TCM) discipline

July 1, 2020

The team list:

Sequence Number	Team Name
1	The Top Team led by Ping Liu
2	The Top Team led by Yuanhua Yang
3	The Top Team led by Guang Ji
4	The Top Team led by Yongqing Yang
5	The Top Team led by Fang Min
6	The Top Team led by Yongjun Wang
7	The Top Team led by Qi Li
8	The Top Team led by Huangan Wu
9	The Gaofeng Gaoyuan Innovation Team led by Xueyong Shen
10	The Gaofeng Gaoyuan Innovation Team led by Wangyi Qin
11	The Gaofeng Gaoyuan Innovation Team led by Xiao Chen
12	The Gaofeng Gaoyuan Innovation Team led by Xuezhong Gong
13	The Gaofeng Gaoyuan Innovation Team led by Yueyi Deng
14	The Gaofeng Gaoyuan Innovation Team led by Huafa Que
15	The Gaofeng Gaoyuan Innovation Team led by Liu Sheng
16	The Gaofeng Gaoyuan Innovation Team led byYongqing Cao
17	The Gaofeng Gaoyuan Innovation Team led by Yuelong Cao
18	The Gaofeng Gaoyuan Innovation Team led by Yueqiu Gao
19	The Gaofeng Gaoyuan Innovation Team led by Chenghai Liu
20	The Gaofeng Gaoyuan Innovation Team led by Yiyang Hu
21	The Gaofeng Gaoyuan Innovation Team led by Wei Yang
22	The Gaofeng Gaoyuan Innovation Team led by Huimin Zhang
23	The Gaofeng Gaoyuan Innovation Team led by Ling Xu
24	The Gaofeng Gaoyuan Innovation Team led by Bin Li
25	The Gaofeng Gaoyuan Innovation Team led by Zhenyi Wang
26	The Gaofeng Gaoyuan Innovation Team led by Wenwei Zhu
27	The Gaofeng Gaoyuan Innovation Team led by Jiatuo Xu

Notice on the approval and related matters of the project funded by the National Natural Science Foundation of China

Dear Mr Xuezhong Gong:

In accordance with the *Regulations of the National Natural Science Foundation of China (NSFC)* and the opinions of experts, NSFC decides to fund the project you have applied for. Project approval identifier: No.81873280. Project name: Study on the pathogenesis of contrast-induced acute kidney injury and the renoprotective mechanism of prepared Dahuang-Chuanxiong from the mitophagy mediated by BINP3 and PINK1/Parkin and mitochondrial quality control. Direct costs: 620,000 Yuan. Project duration: January 2019 to December 2022. The modification and review comments of the project are attached.

Please visit the Internet-based Science Information System (https://isisn.nsfc.gov.cn) as soon as possible to obtain the NSFC Project Proposal and fill in as required. For the projects with modification suggestions, please adjust the relevant contents of the plan in time according to the modification suggestions. Any objection to the modification suggestions should be raised to the relevant office before the deadline for submission of the electronic version of the project plan.

The electronic version of the project plan should be uploaded to the Internet-based Science Information System (https://isisn.nsfc.gov.cn). After verification by the supporting institution, it shall be submitted to NSFC for further examination. Those who fail to pass the examination shall be submitted after revising. The applicant who has passed the examination shall print the plan (in two copies, printed on both sides), which shall be examined and stamped by the supporting institution. And then the above documents shall be submitted to the Project Materials Receiving Working Group of NSFC. The electronic version of the project plan should be consistent with the printed version.

The deadline for resubmitting the signature and seal page of the printed application form, uploading and submitting the project plan to NSFC:

1. The deadline for uploading the electronic version of the project plan (regarded as the official submission time) is **16:00 on September 11, 2018**;

2. The deadline for uploading the electronic revised project plan is **16:00 on September 18, 2018**;

3. The deadline for submitting the printed project plan is **16:00 on September 26, 2018**.

Please uploading the electronic version of the project plan in time according to the above provisions, and submit the printed version. Those who fail to submit the plan or the signature

and seal page of the printed application form before the deadline without giving reasons will be deemed to automatically give up accepting the funding.

Attachment: Project Review Comments and Modification Suggestions Table

National Natural Science Foundation

Medical Sciences Division

August 16, 2018

Notice on the approval and related matters of the project funded by the National Natural Science Foundation of China

Dear Mr Xuezhong Gong:

In accordance with the *Regulations of the National Natural Science Foundation of China (NSFC)* and the opinions of experts, NSFC decides to fund the project you have applied for. Project approval identifier: No.82074387. Project name: Study on the pathogenesis of trivalent arsenic induced kidney injury and the renal protective mechanisms of Chuanhuang Fang from GPX4, p62/Nrf2/HO-1-mediated ferroptosis of renal tubular epithelial cells. Direct costs: 550000 Yuan. Project duration: January 2021 to December 2024. The modification and review comments of the project are attached.

Please visit the Internet-based Science Information System (https://isisn.nsfc.gov.cn) as soon as possible to obtain the NSFC Project Proposal and fill in as required. For the projects with modification suggestions, please adjust the relevant contents of the plan in time according to the modification suggestions. Any objection to the modification suggestions should be raised to the relevant office before the deadline for submission of the electronic version of the project plan.

The electronic version of the project plan should be uploaded to the Internet-based Science Information System (https://isisn.nsfc.gov.cn). After verification by the supporting institution, it shall be submitted to NSFC for further examination. Those who fail to pass the examination shall be submitted after revising. The applicant who has passed the examination shall print the plan (in two copies, printed on both sides), which shall be examined and stamped by the supporting institution. And then the signature and seal page of the printed application form shall be affixed to one of the printed documents. Finally, the above documents shall be submitted to the Project Materials Receiving Working Group of NSFC. NSFC will review the signature and seal page of the printed application. If there is any problem, the supporting institution is allowed once to revise or submit it.

The deadline for resubmitting the signature and seal page of the printed application form, uploading and submitting the project plan to NSFC:

1. **16:00 on October 23, 2020:** The deadline for uploading the electronic version of the project plan (regarded as the official submission time);

2. **16:00 on October 30, 2020:** The deadline for uploading the electronic revised project plan;

3. **16:00 on November 6, 2020:** The deadline for submitting the printed project plan (one of which contains the signature and seal page of the printed application form);

4. **16:00 on November 27, 2020:** The deadline for submitting the signature and seal page of the printed revised application form.

Please uploading the electronic version of the project plan in time according to the above provisions, and submit the printed version and the signature and seal page of the printed application form. Those who fail to submit the plan or the signature and seal page of the printed application form before the deadline without giving reasons will be deemed to automatically give up accepting the funding. If the applicant fails to revise the application form as required or fails to submit the signature and seal page of the application form in time, he/she will be postponed the appropriation of funds according to the circumstances.

Attachment: Project Review Comments and Modification Suggestions Table

National Natural Science Foundation

September 27, 2020
