# Supplementary material for: Clinical efficacy of Tailin formulation combined with continuous low-dose antimicrobial therapy for recurrent urinary tract infection: study protocol for a multicenter, double-blind, randomized, controlled clinical trial
Source: Trials. 2021 Dec 11;22:901. doi: 10.1186/s13063-021-05830-4 (PMC8665585; doi:10.1186/s13063-021-05830-4)
Supplement: Supplementary file 4 — Additional file 4. The informed consent document. [file 13063_2021_5830_MOESM4_ESM.pdf]

知情同意书•知情告知页

尊敬的先生/女士：

我们将邀请您参加一项“泰淋方联合抑菌疗法中西医结合方案治疗复发性尿路感染随机、对照、多中心临床研究”。

在您决定是否参加这项研究之前，请尽可能仔细阅读以下内容，它可以帮助您了解该项研究以及为何要进行这项研究，研究的程序和期限，参加本研究后可能给您带来的益处、风险和不适。如果您愿意，您也可以和您的亲属、朋友一起讨论，或者请您的医生给予解释，帮助您做出决定。

研究介绍

尿路感染是一种临床常见疾病，容易复发，即便感染控制以后，患者的非感染性症状（比如腰背酸痛、下腹坠胀）可长时间存在、难于消除，给患者带来极大痛苦。临床上一年内发作 3 次及以上的尿路感染被称为复发性尿路感染。

长程、低剂量抗生素疗法（即抑菌疗法）是西医治疗复发性尿路感染的首选方法。但其疗效有限，潜在的副作用不容忽视，包括肝肾损伤风险、耐药菌株出现、增加真菌感染几率、胃肠道损伤等。为改善该疾病的治疗现状，寻找一个更加有效、安全的治疗方法，我们开展了本研究。

复发性尿路感染属于中医“劳淋”范畴，采用中医中药治疗“淋证”具有悠久历史。课题组多年来一直从事 rUTI 相关临床和实验研究,拟定了治疗 rUTI 的方剂泰淋方。该方主要由太子参、生地、（怀）牛膝、生米仁等“药食同源”的中药组成。

本研究将在 3 个研究中心进行，预计共有 200 名受试者自愿参加。

本研究项目为上海市教科委高峰高原学科建设项目（课题编号：No.02.ZY05.191311N ）。本研究项目由上海市中医医院伦理委员会审议，遵从中国国家相关法规和赫尔辛基宣言等保护受试者权益的伦理原则。

二、入选标准和排除标准

入选标准：

①符合复发性尿路感染诊断标准

②符合中医脾肾气阴亏虚、湿热流恋型劳淋辨证诊断标准；

③年龄 18 至 70 岁，性别不限；

④能够理解试验的全过程，自愿参加并签署知情同意书。

排除标准：

①新发急性尿路感染；

②留置导尿管；

③有泌尿结构或功能异常，如泌尿道先天畸形、肾盂结石或其他病变；

④尿道综合征；

⑤CKD IV-V 期(eGFR<30ml/min)；

⑥合并严重心肝功能损害或糖尿病等需要立即治疗的疾病；

⑦对本研究的两种及以上抗生素过敏；

⑧怀孕或哺乳期女性(育龄妇女需做尿 HCG 检测)；

⑨患有严重的中枢神经系统疾病；

⑩最近 3 个月内有参加其他药物临床试验或参加过其他药物临床试验。

三、如果参加研究将需要做什么？

1、如果您符合入选标准并同意参加，将按以下步骤进行研究：

入组治疗前我们会对您进行体格检查、生命体征及症状相关的检查；采集您的血液及尿液样本进行尿沉渣、尿常规、清洁中段尿培养+药敏+计数、尿 NAG/尿肌酐、尿β2-MG、尿α1-MG、尿转铁蛋白、尿 HCG 试验（育龄妇女）、心电图、血常规、尿常规、粪常规+粪隐血、肝功能(GOT、GPT)、肾功能(BUN、Scr)等项目的检测。

若无排除选项，将随机予中药或模拟剂联合长程低剂量抗生素疗法治疗 12 周，每 2 周随访一次，作必要项目及体征症候评估，随访至 24 周。若期间出现意外情况可随时终止试验。

2. 需要您配合的其他事项：无

四、参加研究的受益

您可能会得到很好的控制和治疗，改善生活水平和质量。您的参加将对研究治疗复发性尿路感染提供新方法。将有助于对疾病作出诊断，为您的治疗提供必要的建议，或为疾病的研究提供有益的信息。

潜在受益：本研究可能会阻止/减缓疾病的发展，但是我们不能对此做出保证。尽管参加本次研究可能不会给您带来直接的益处，但您的参加可能会给未来遭受同样痛苦的患者带来益处。

五、参加研究的风险

本研究所用的泰淋方颗粒，由“药食同源”的中药组成，目前未有不良反应发生，少数患者可能会有轻微胃部不适等。联合使用的长程低剂量抗生素疗法（抑菌疗法），包括三种指定抗生素（左旋氧氟沙星、呋喃坦啶片、头孢地尼）及两种备用抗生素（复方新诺明、磷霉素丁三醇）。通过对相关报道进行统计，抑菌疗法的不良反应可能有（1）全身：腹痛、乏力/疲劳、胸痛、水肿/肿胀。（2）心血管系统：心悸、心动过速。（3）消化系统：腹泻、消化不良、恶心。（4）肌肉骨骼系统：肌痛、肌肉筋挛。（5）神经/精神系统：头晕、头痛、失眠。（6）呼吸系统：咳嗽、鼻充血、咽炎、上呼吸道感染。（7）过敏症：偶有浮肿、荨麻疹、皮疹、瘙痒、红斑等症状。（8）肾脏：偶见血中尿素氮升高。（9）肝脏：可能出现一过性肝功能异常，如血清总胆红素增加等。

参加该试验科可能会对您的病情有帮助，也可能会不能遏制病情发展使病情进一步加重。以上风险需要谨慎。在研究期间，也许会出现其他一些不适，请立即告诉您的研究医师，他/她会对您出现的不适进行判断和医疗处理。

研究中您需按照研究者的要求时间点来访视，可能会对您带来不便。

六、参加研究的费用、补偿和赔偿

参加本研究使用的药物和相关检查（尿沉渣、尿常规、清洁中段尿培养+药敏+计数、尿 NAG/尿肌酐、尿β2-MG、尿α1-MG、尿转铁蛋白、尿 HCG 试验（育龄妇女）、心电图、血常规、尿常规、粪常规+粪隐血、肝功能(GOT、GPT)、肾功能(BUN、Scr)）是免费的。经专家委员会认定，您确实发生了与本研究有关的损伤，申办者/课题组将按照国家法律法规承担相应责任，并对试验相关的损害给予相应的补偿或赔偿。

如果您同时合并其他疾病所需的治疗和检查，将不在免费的范围之内。

七、个人信息是保密的吗？

您参加本项研究的信息均会记录在研究病历/病例报告表中。所有出现在原始医学记录中的研究结果（包括个人资料、化验单据等）均会在法律的允许范围内完全保密。您的名字不会出现在 CRF 表中，仅仅出现您的姓名拼音缩写和在您参加研究时分配的编号。相关研究总结、文章、公开刊物中，如有必要，也只会仅出现您的姓名拼音缩写和编号。

必要时，药品监督管理部门、伦理委员会或课题资助部门，按规定可以查阅参加研究的受试者资料。但未经允许，他们不会将参加研究的受试者资料用到其他的用途或泄露给其他的团体。

八、怎样获得更多的信息？

您可以在任何时间提出有关本项研究的任何问题。

您的医生将给您留下他/她的电话号码以便能回答您的问题。

如果在研究过程中有任何重要的新信息，可能影响您继续参加研究的意愿时，您的医生将会及时通知您。

九、可以自愿选择参加研究和中途退出研究

是否参加本研究完全取决于您的自愿。您可以拒绝参加此项研究，或在研究过程中的任何时间退出本研究。如果您选择退出此研究，您的受益将不会受到影响，也不会因此而受到歧视或报复。

您的医生或研究者出于对您的最大利益考虑，可能会随时中止您参加本项研究。

如果您因为任何原因从研究中退出，您可能被咨询有关您使用研究药物的情况。如果医生认为需要，您也可能被要求进行实验室检查和体格检查。您也可以拒绝，并不会因此受到歧视或报复。

如果您选择参加本项研究，我们希望您能够坚持完成全部研究过程。

十、现在该做什么？

是否参加本项研究由您自己决定。您可以和您的家人或者朋友讨论后再做出决定。

在您做出参加研究的决定前，请尽可能向您的医生询问有关问题，直至您对本项研究完全理解。

十一、伦理委员会

如果您有疑问或需要向除研究者以外的人员询问，请咨询上海市中医医院伦理委员会。

伦理委员会办公室： 上海市中医医院九号楼二楼　　伦理委员会办公室

联系电话：021-56628310

感谢您阅读以上材料。如果您决定参加本项研究，请告诉您的医生，他/她会为您安排一切有关研究的事务。

请您保留这份资料。

知情同意书•同意签字页

项目名称：泰淋方联合抑菌疗法中西医结合方案治疗复发性尿路感染随机、对照、多中心临床研究

版本日期：2021 年 04 月 20 日

同意声明

我已经阅读了上述有关本研究的介绍，而且有机会就此项研究与医生讨论并提出问题。我提出的所有问题都得到了满意的答复。

我知道参加本研究可能产生的风险和受益。我知晓参加研究是自愿的，我确认已有充足时间进行考虑，而且明白：

- 我可以随时向医生咨询更多的信息。
- 我可以随时退出本研究，而不会受到歧视或报复，医疗待遇与权益不会受到影响。

我同样清楚，如果我中途退出研究，特别是由于药物的原因使我退出研究时，我若将病情变化告诉医生，完成相应的体格检查和理化检查，这将对我本人和整个研究十分有利。

如果因患病的需要采取任何其他的药物治疗，我会在事先征求医生的意见，或在事后如实告诉医生。

我同意药品监督管理部门、伦理委员会或课题资助部门代表查阅我的研究资料。

我将获得一份经过签名并注明日期的知情同意书副本。

最后，我决定同意参加本项研究，并保证尽量遵从医嘱。

受试者签名：__________________ 日期：_______ 年 _____ 月_____日

受试者联系电话：___________________

我确认已向受试者解释了本研究的详细情况，包括其权利以及可能的受益和风险，并给其一份签署过的知情同意书副本。

研究者签名：____________________ 日期：_______ 年______ 月_____日

研究者联系电话：__________________________
